# Analysis of SMARCA4 and SMARCA2 Loss in Lung Sarcomatoid Carcinomas*

**DOI:** 10.5146/tjpath.2022.01590

**Published:** 2023-05-15

**Authors:** Halide Nur Urer, Nurcan Unver, Neslihan Fener

**Affiliations:** Department of Pathology, University of Health Sciences Turkey, Haseki Training and Research Hospital, Istanbul, Turkey; Yedikule Chest Diseases and Thoracic Surgery Training and Research Hospital, Istanbul, Turkey

**Keywords:** Lung neoplasm, Carcinosarcoma, Pleomorphic, Pulmonary blastoma, SMARCA4, SMARCA2

## Abstract

***Objective:*** Sarcomatoid carcinomas of the lung are a group of aggressive tumors. It has been reported that losses of SMARCA4 and SMARCA2, which play a role in the repair and remodeling of chromatin, contribute to the initiation, progression, and differentiation of neoplasms. The aim of our study was to examine SMARCA4 and SMARCA2 profiles in sarcomatoid carcinomas of the lung.

***Material and Method:*** We screened pleomorphic carcinomas (PCs), carcinosarcomas (CSs), and pulmonary blastomas (PBs). The loss of SMARCA4 and SMARCA2 expression in the tumors was evaluated using immunohistochemical methods. The tumors were also examined to determine immunophenotype, histological tumor diagnosis, surgical resection, tumor histological component, largest tumor diameter, and lymph node metastasis status.

***Results:*** Sixty-nine cases were screened, of which 84% were PCs, 13% were CSs, and 2.8% were PBs. In PCs components, 84.4% were biphasic and 15.5% were monophasic. The PCs showed the most frequent loss of SMARCA4 (25.8%) and SMARCA2 (44.8%). A loss of SMARCA4 and SMARCA2, respectively, was detected in 14.2% and 24.4% in both components of biphasic PCs; 12.2% and 14.2% in the sarcoma component of biphasic PCs; 0% and 8.1% in the carcinoma component of biphasic PCs; 22.2% and 33.3% in monophasic PCs; 0% and 22.2% in both components of CSs; and 0% and 22.2% in the sarcoma component of CSs.

***Conclusion:*** These findings demonstrate a loss of expression of SMARCA4 and SMARCA2 in pulmonary sarcomatoid carcinomas. Loss of the SMARCA complex may be caused by the heterogeneous morphological profile of sarcomatoid carcinomas, independent of tumor histopathological parameters.

## INTRODUCTION

Sarcomatoid carcinomas of the lung are a group of aggressive organotrophic tumors containing heterologous elements. This group includes pleomorphic carcinomas (PCs), carcinosarcomas (CSs), and pulmonary blastomas (PBs) with sarcoma and carcinoma components. These tumors typically exhibit high epithelial-mesenchymal transformation abilities. Because of their diversity and heterogeneity, their biological behavior generally includes nodal lymph node involvement and distant metastasis.

The Switch/Sucrose Nonfermenting (SWI/SNF) complex is a family of proteins involved in chromatin remodeling (1). A subunit of the complex, SMARCA4 (BRG1), is encoded in the 19p13.2 chromosome region and is involved in the ATP-dependent transcriptional regulation process. SMARCA2 (BRM) is the other major catalytic subunit of the SWI/SNF family. The SWI/SNF protein complex is active in the initiation, progression, and differentiation of neoplasms. It is active in the pathogenetic pathway of complex, undifferentiated, and aggressive pediatric tumors with a rhabdoid phenotype, malignant rhabdoid tumors of the kidney, extrarenal soft tissue tumors, and visceral organ tumors (2–4). In addition, specific neoplasms in the gastrointestinal, pancreatic, sinonasal, and genitourinary systems are characterized by a loss of subunits (2)**. **Yoshida et al. suggest thoracic undifferentiated sarcoma and carcinomas with rounded or epithelioid histology and loss of SMARCA as a separate group of tumors (5). These are highly aggressive tumors. On the other hand, the *SMARCA4 *alteration rate is 2.7-10% in common carcinomas of the lung such as adenocarcinoma, small cell carcinoma, squamous cell carcinoma, non-small cell carcinoma, and large cell neuroendocrine carcinoma (6). Loss of the complex is an important finding in both poorly differentiated/undifferentiated carcinomas and undifferentiated sarcomas of the thorax. However, data on the *SMARCA* profile and phenotypic features in lung sarcomatoid carcinomas are quite limited.

Our study investigated the morphological features of sarcomatoid carcinomas of the lung. We evaluated the loss of SMARC4A and SMARCA2 in this heterogeneous tumor group. The relationship of protein loss with tumor components and histopathological prognostic factors was examined. Our aim was to determine the features of SMARCA4 and SMARCA2 deficiency in lung sarcomatoid carcinomas.

## MATERIALS and METHODS

In the present study, we searched for patients diagnosed with PCs, CSs, or PBs who underwent anatomic lung tumor resection between January 1, 2010, and December 31, 2019. The cases identified were re-evaluated according to the sarcomatoid carcinoma criteria of the World Health Organization Lung Tumor Classification. Surgical nonanatomical resections, sarcomatoid carcinoma metastases of extrapulmonary organs, and cases for which specimens could not be obtained were excluded. Age, sex, histological tumor type, surgical resection type, tumor histological component, largest tumor diameter, and lymph node metastasis status were investigated. Ethics committee approval was obtained for the study (2258/2020).

### Immunohistochemical Analysis

For each case, we selected formalin-fixed paraffin-embedded tumor blocks containing carcinoma and sarcoma components. Tissue samples were taken from one or two paraffin blocks for each case. These samples were mapped and new paraffin blocks were prepared, each containing 14 tissues. Three-micron sections were taken from the blocks. Pancytokeratin by immunohistochemistry (CAM5.2; Master Diagnostica, Dubai, UAE), TTF-1(8G7G3/; Santa Cruz Biotechnology, Dallas, TX, USA), P40 (ZR8; Master Diagnostica), E-cadherin (BS38; Master Diagnostica), SMARCA4 antibodies (orb513921; Abcam, Cambridge, UK), SMARCA2 antibodies (orb575109; Abcam), and Ki-67 (SP6; Master Diagnostica) were applied. All staining was performed on a Ventana fully automated immunohistochemistry device (Roche Diagnostics, Basel, Switzerland). We used the universal kit Ventana BenchMark XT and BenchMark ULTRA instruments (Roche Diagnostics).

For immunohistochemical evaluation, pancytokeratin staining was categorized as diffuse, focal, or absent (+2/+1/-); TTF-1 as diffuse, focal, or absent (+2/+1/-); P40 as 50% or more, less than 50%, or absent (+2/+1/-); E-cadherin as diffuse, focal, or absent (+2/+1/-); and Ki-67 nuclear staining as over 50%, 10-50%, or less than 10% (2/1/0). SMARCA4 and SMARCA2 were evaluated as ‘1’ if there was any positive nuclear expression and ‘0’ if none. For pancytokeratin, TTF-1, and E-cadherin, we considered the results positive if diffuse or focal staining was present. For P40, staining of 50% or more was considered positive.

### Statistical Analysis

Data were analyzed using descriptive statistics. An analysis was carried out using the SPSS 25 package program. Relationships between two categorical variables were examined with the chi-square test. The Yates correction and p-values were used when appropriate. In the study, the

The p-value was considered significant if it was found to be less than 0.05.

## RESULTS

A total of 70 sarcomatoid carcinoma cases were identified. One case was excluded because specimens could not be obtained. A total of 131 samples were taken from formalin-fixed, paraffin-embedded blocks for the remaining 69 cases. The age range of the cases was 34–76, the mean age was 60.8 years, and the median age was 61 years. The complete demographic characteristics, type of surgical intervention, largest tumor size, and lymph node metastasis status are shown in Table 1. The tumor diameter range was 2.1–18 cm.

**Table 1:** Characteristics of cases.

**Features**	**Number of cases (%), n = 69**
Histological type	
Pleomorphic carcinomas	58 (84)
Carcinosarcomas	9 (13)
Pulmonary blastomas	2 (2.8)
Sex	
Male	62 (89.8)
Female	7 (10.1)
Surgical intervention	
Lobectomy	44 (63.7)
Bilobectomy	2 (2.8)
Pneumonectomy	20 (28.9)
Tumor diameter in centimeters	
2 ≤ diameter < 3	9 (13)
3 ≤ diameter < 4	8 (11.5)
4 ≤ diameter < 5	8 (11.5)
5 ≤ diameter < 7	23 (33.3)
7 < diameter	21 (30.4)
Lymph node metastasis	
N0	45 (65.2)
N1	17 (24.6)
N2	7 (10.1)

**N0:** No lymph node metastasis, **N1:** Ipsilateral intrapulmonary lymph node metastasis, **N2:** Ipsilateral mediastinal lymph node metastasis.

**Figure 1:**
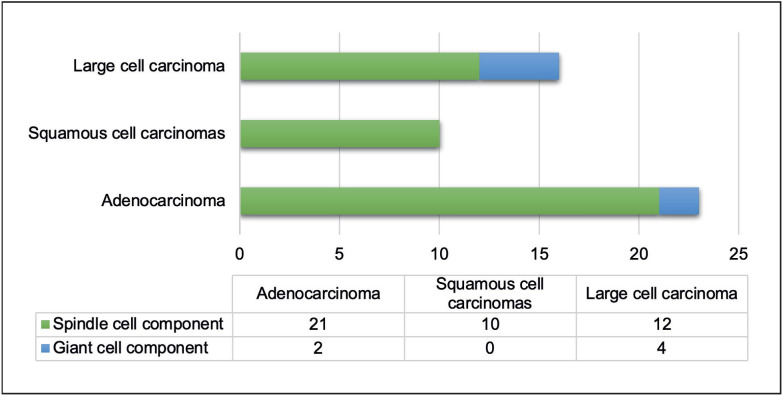
Distribution of biphasic pleomorphic carcinomas with carcinoma and sarcoma components.

Of the 58 PCs cases, 49 (84.4%) were biphasic and 9 (15.5%) were monophasic. The carcinoma and sarcoma components of biphasic cases were evaluated (Figure 1). Their distribution was as follows: 46.9% adenocarcinoma, 20.4% squamous cell carcinoma, and 32.6% large cell carcinoma. Overall, 87.7% of the sarcoma components of the PCs were spindle cells and 12.2% giant cells.

Of the remaining nine monophasic PCs, five were pure spindle cells and four were pure giant cells.

Among the CS cases, we observed 66.6% squamous cell carcinomas, 22.2% adenocarcinomas, and 11.1% large cell carcinomas. The dominant type among the sarcoma components was chondrosarcoma (55%) (Table 2).

**Table 2:** Tumor components of carcinosarcomas.

**Tumor component**	**Chondrosarcoma**	**Osteosarcoma**	**Synovial sarcoma**	**Rhabdomyosarcoma**	**Total**
Adenocarcinoma	1	-	1	-	2
Squamous cell carcinoma	3	2	-	1	6
Large cell carcinoma	1	-	-	-	1
Total	5	2	1	1	9

Our immunohistochemical results for the sarcomatoid carcinoma cases are shown in Table 3.

**Table 3:** Immunohistochemical results for sarcomatoid carcinomas.

**Tumor**	**Histologic type**	**PCK (+2/+1/-)**	**TTF-1 (+2/+1/-)**	**P40 (+2/+1/-)**	**E-cadherin (+2/+1/-)**	**Ki-67 (+2/+1/-)**	**SMARCA4 (1/0)**	**SMARCA2 (1/0)**
PCs biphasic	SC (10)	6/4/0	0/0/10	7/2/1	4/5/1	3/6/1	9/1	8/2
	AC (23)	23/0/0	15/5/3	0/1/0	9/11/3	9/12/2	23/0	15/8
	LCC (16)	14/1/1	1/0/15	1/1/14	1/7/6	11/4/1	10/6	10/6
	Spindle cell component (43)	15/16/12	4/4/35	1/6/36	1/4/38	14/23/6	32/11	26/17
	Giant cell component (6)	3/2/1	1/1/4	0/0/6	1/0/5	2/3/1	4/2	3/3
PCs monophasic	Spindle cell carcinoma (5)	4/1/0	1/0/4	0/0/5	0/2/3	0/4/1	4/1	4/1
	Giant cell carcinoma (4)	4/0/0	1/0/3	0/0/4	0/1/3	1/2/1	3/1	3/1
CSs	SC (6)	1/2/3	0/0/6	2/3/1	2/3/1	0/6/0	5/1	4/2
	AC (2)	1/1/0	0/1/1	0/0/2	1/0/1	0/2/0	2/0	0/2
	LCC (1)	1/0/0	0/0/1	0/0/1	0/1/0	0/1/0	1/0	1/0
	Chondrosarcoma (5)	0/1/4	0/0/5	0/0/5	0/0/5	0/5/0	5/0	5/0
	Osteosarcoma (2)	0/0/2	0/0/2	0/0/2	0/0/2	0/2/0	2/0	1/1
	Synovial sarcoma (1)	1/0/0	0/0/1	0/0/1	0/0/1	0/1/0	1/0	0/1
	Rhabdomyosarcoma (1)	1/0/0	0/0/1	0/0/1	0/0/1	0/1/0	1/0	1/0
PBs	Epithelial component (2)	0/0/2	0/1/1	0/1/1	0/0/2	0/2/0	2/0	2/0
	Sarcoma component (2)	0/0/2	0/0/2	0/0/2	0/0/2	1/1/0	2/0	2/0

**PCs:** Pleomorphic carcinoma, **CSs:** Carcinosarcoma, **PBs:** Pulmonary blastoma, **SC:** Squamous cell carcinoma, **AC:** Adenocarcinoma, **LCC:** Large cell carcinoma.

**Figure 2:**
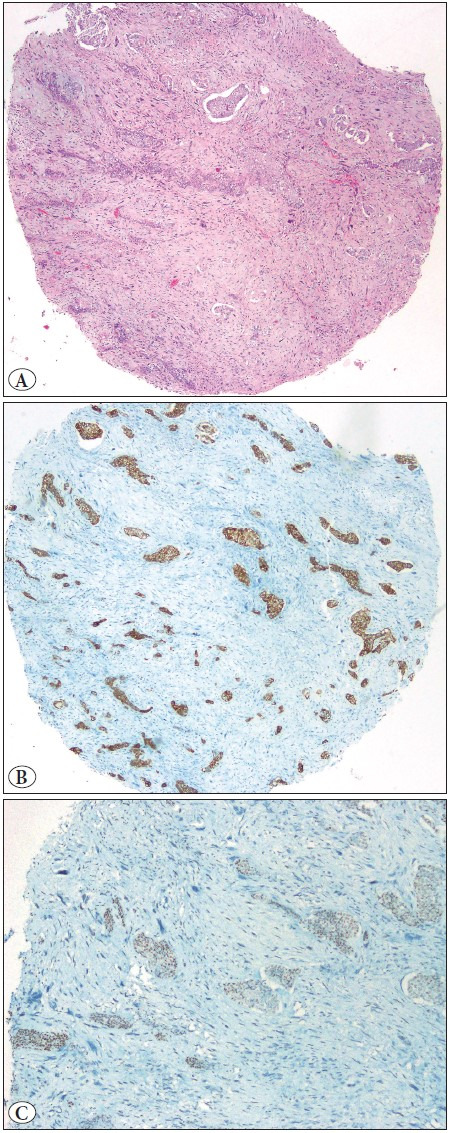
**A)** Spindle cell and squamous cell pleomorphic carcinomas, **B)** Expression of pancytokeratin. Squamous cell carcinoma component-positive, spindle cell componentnegative, **C)** SMARCA4 appears to be preserved in the squamous component and missing from the spindle component.

The distribution of SMARCA4 and SMARCA2 loss in the tumor components of sarcomatoid carcinomas is shown in Table 4. SMARCA2 loss was greater than SMARCA4 loss. The highest loss was detected in PCs (Figure 2).

**Table 4:** Features of SMARCA4 and SMARCA2 deficiency.

**Tumors**	**Components**	**SMARCA4 deficiency (%)**	**SMARCA2 deficiency (%)**
PCs	Total	15 (25.8)	26 (44.8)
	Biphasic PCs, loss of both components	7 (14.2)	12 (24.4)
	Biphasic PCs, loss of the sarcoma component	6 (12.2)	7 (14.2)
	Biphasic PCs, loss of the carcinoma component	0 (0)	4 (8.1)
	Monophasic PCs	2 (22.2)	3 (33.3)
CSs	Total	1 (11.1)	4 (44.4)
	CSs, loss of both components	0 (0)	2 (22.2)
	CSs, loss of the sarcoma component	0 (0)	2 (22.2)
	CSs, loss of the carcinoma component	1(11.1)	2 (22.2)

**PCs:** Pleomorphic carcinomas, **CSs:** Carcinosarcomas.

**Figure 3:**
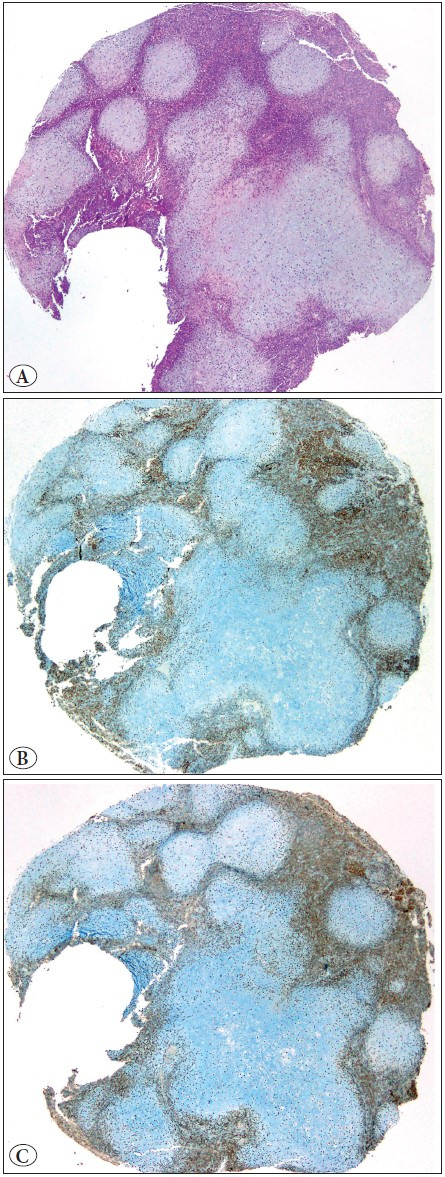
**A)** Carcinosarcoma with a chondrosarcoma component, **B)** Preserved SMARCA4 expression in a carcinosarcoma, **C)** Preserved SMARCA2 expression in a carcinosarcoma.

The loss in CSs was 22.2–33.3% (Figure 3).

**Figure 4:**
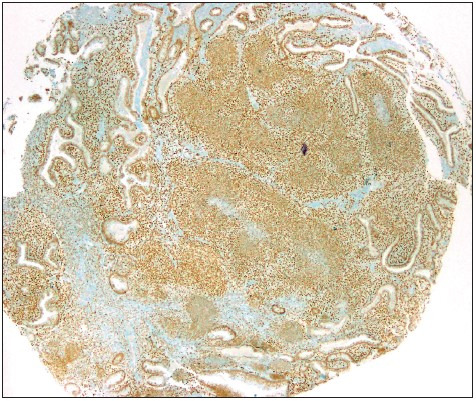
Preserved SMARCA4 expression in a pulmonary blastoma.

No loss was observed in two cases with PB (Figure 4).

A total of 12 (20.6%) cases showing a loss of both SMARCA4 and SMARCA2 were identified; 11 (18.9%) showed a loss of the same component. One showed a loss in a different component. All of these tumors were biphasic PCs.

We explored prognostic histopathological parameters and their relationship with histological type in SMARCA-deficient tumors (Table 5). There were no significant differences in tumor diameter, lymph node metastasis status, or tumor histology.

**Table 5:** SMARCA deficiency and tumor prognostic parameters.

**Tumor features**	**SMARCA4 deficiency n=16 (%)**	**NonSMARCA4 deficiency n=53 (%)**	**p** **value**	**SMARCA2 deficiency n=30 (%)**	**NonSMARCA2 deficiency n=39 (%)**	**p** **value**
Tumor diameter						
0–3cm >3cm	3 (18.7) 13 (81.2)	6 (11.3) 47 (88.6)	0.72	3 (10) 27 (90)	6 (15.3) 33 (84.6)	0.76
0–5cm >5cm	8 (50) 8 (50)	17 (32) 36 (67.9)	0.31	8 (26.6) 22 (73.3)	17 (43.5) 22 (56.4)	0.23
Lymphnodemetastasis						
N0 N1+N2	13 (81.2) 3 (18.7)	32 (60.3) 21 (39.6)	0.21	18 (60) 12 (40)	27 (69.2) 12 (30.7)	0.58
Histologictype						
PCs CSs+PBs	15 (93.7) 1 (6.2)	43 (81.1) 10 (18.8)	0.41	26 (86.6) 4 (13.3)	32 (82) 7 (17.9)	0.85

## DISCUSSION

In our study, a loss of SMARCA4 and SMARCA2 in pulmonary sarcomatoid carcinoma was found in 23.1% and 43.4% of cases, respectively. Deficient expression was high in PCs, but not in PBs. The deficiency of the complex in lung PCs varies from 36% to 41% (7). While subunit silencing is seen in differentiated non-small cell lung carcinomas, increased epithelial-mesenchymal transformation may be responsible for the aggressive character of sarcomatoid carcinomas.

Sarcomatoid carcinomas are rare malignant pulmonary tumors (8). They are heterogeneous neoplasms with different variants and components with epithelial and non-epithelial mesenchymal phenotypes. PCs were the most frequently detected type of sarcomatoid carcinoma in our study. This finding is consistent with the available data (9). However, squamous cell carcinoma is the most common type of carcinosarcoma and large cell carcinoma is the least common. Chondrosarcoma was the most common type in the malignant mesechymal component. In the pleomorphic carcinoma study of A.W. et al., adenocarcinoma was detected most frequently in both the dedifferentiated and sarcomatoid elements (10). Chondrosarcoma was the most common type in the malignant mesechymal component. The diversity of subgroups in pleomorphic carcinoma may cause difficulties in classifying these tumors. The difference in the loss of SMARCA4 and SMARCA2 in these subgroups should be investigated in larger studies.

A concomitant loss of SMARC subunits in different tumors has been described. A loss of both SMARCA4 and SMARCA2 has been found in 10–26% of non-small cell carcinomas (11–13). The mutual inactivation of only SMARCA2-SMARB1 was detected in gastrointestinal undifferentiated carcinomas with a rhabdoid phenotype (14). Similarly, a loss of multiple SMARC subunits was detected in PCs with biphasic components in the present study. Concomitant inactivation has been reported to have a negative effect on survival, independent of tumor stage (11).

The SWI/SNF complex plays an important role in tumor suppression and DNA repair by remodeling chromatin. The most important subunits of the complex include SMARCA4, SMARCA2, SMARCB1, ARID1A, ARID1B, SMARCC2, SMARCE1, SMARCD1/2/3, BRD9, BCL7, BCL11A/B, DPF2, and SS18 (15). Monoallelic or biallelic SMARCA loss is often associated with tumor dedifferentiation and/or rhabdoid morphology (16,17). A thoracic SMARCA4-deficient undifferentiated tumor (also known as a SMARCA4-deficient thoracic sarcoma) is a newly identified SMARCA-deficient neoplasm with an ICD-O code (6). The tumor is a high-grade malignancy with an undifferentiated and/or rhabdoid phenotype. However, inactivation is found in various tumors. Examples include soft tissue tumors, head and neck tumors, endometroid and gastrointestinal carcinomas, and particularly malignancies with undifferentiated and rhabdoid morphologies (14,16,18). Conversely, it has been suggested that the rhabdoid phenotype may not be associated with SMARCA loss in dedifferentiated or undifferentiated carcinomas (19). Similarly, unit losses without a rhabdoid morphology have been reported in a limited case series of lung carcinomas (20). Thoracic tumors with rhabdoid features may not be mandatory for SMARC deficiency.

## CONCLUSION

Pulmonary sarcomatoid carcinomas are neoplasms with a loss of SMARCA4 and SMARCA2 expression. A loss of SMARC may result from the heterogeneous morphological profile of sarcomatoid carcinomas, independent of histopathological parameters.

## Funding

This study was financially supported as a Project (no: 2020/052) in the Scientific Research Program of the University of Health Sciences, Turkey.

## Conflict of Interest

As the authors of our manuscript, we declare that there is no conflict of interest regarding the publication of this paper

## References

[ref-1] Lu Ping, Roberts Charles W. M. (2013). The SWI/SNF tumor suppressor complex: Regulation of promoter nucleosomes and beyond. Nucleus.

[ref-2] Pawel Bruce R. (2018). SMARCB1-deficient Tumors of Childhood: A Practical Guide. Pediatr Dev Pathol.

[ref-3] Halliday Gary M., Bock Vanessa L., Moloney Fergal J., Lyons J. Guy (2009). SWI/SNF: a chromatin-remodelling complex with a role in carcinogenesis. Int J Biochem Cell Biol.

[ref-4] Agaimy Abbas, Bertz Simone, Cheng Liang, Hes Ondrej, Junker Kerstin, Keck Bastian, Lopez-Beltran Antonio, Stöckle Michael, Wullich Bernd, Hartmann Arndt (2016). Loss of expression of the SWI/SNF complex is a frequent event in undifferentiated/dedifferentiated urothelial carcinoma of the urinary tract. Virchows Arch.

[ref-5] Yoshida Akihiko, Kobayashi Eisuke, Kubo Takashi, Kodaira Makoto, Motoi Toru, Motoi Noriko, Yonemori Kan, Ohe Yuichiro, Watanabe Shun-Ichi, Kawai Akira, Kohno Takashi, Kishimoto Hiroshi, Ichikawa Hitoshi, Hiraoka Nobuyoshi (2017). Clinicopathological and molecular characterization of SMARCA4-deficient thoracic sarcomas with comparison to potentially related entities. Mod Pathol.

[ref-6] Sesboue Come, Le Loarer Francois (2021). SWI/SNF-deficient thoraco-pulmonary neoplasms. Semin Diagn Pathol.

[ref-7] Yoshimoto Taichiro, Matsubara Daisuke, Nakano Tomoyuki, Tamura Tomoko, Endo Shunsuke, Sugiyama Yukihiko, Niki Toshiro (2015). Frequent loss of the expression of multiple subunits of the SWI/SNF complex in large cell carcinoma and pleomorphic carcinoma of the lung. Pathol Int.

[ref-8] Travis William D. (2010). Sarcomatoid neoplasms of the lung and pleura. Arch Pathol Lab Med.

[ref-9] Rossi Giulio, Cavazza Alberto, Sturm Nathalie, Migaldi Mario, Facciolongo Nicola, Longo Lucia, Maiorana Antonio, Brambilla Elisabeth (2003). Pulmonary carcinomas with pleomorphic, sarcomatoid, or sarcomatous elements: a clinicopathologic and immunohistochemical study of 75 cases. Am J Surg Pathol.

[ref-10] Weissferdt Annikka, Kalhor Neda, Correa Arlene M., Moran Cesar A. (2017). "Sarcomatoid" carcinomas of the lung: a clinicopathological study of 86 cases with a new perspective on tumor classification. Hum Pathol.

[ref-11] Reisman David N., Sciarrotta Janiece, Wang Weidong, Funkhouser William K., Weissman Bernard E. (2003). Loss of BRG1/BRM in human lung cancer cell lines and primary lung cancers: correlation with poor prognosis. Cancer Res.

[ref-12] Matsubara Daisuke, Kishaba Yuka, Ishikawa Shumpei, Sakatani Takashi, Oguni Sachiko, Tamura Tomoko, Hoshino Hiroko, Sugiyama Yukihiko, Endo Shunsuke, Murakami Yoshinori, Aburatani Hiroyuki, Fukayama Masashi, Niki Toshiro (2013). Lung cancer with loss of BRG1/BRM, shows epithelial mesenchymal transition phenotype and distinct histologic and genetic features. Cancer Sci.

[ref-13] Herpel Esther, Rieker Ralf J., Dienemann Hendrik, Muley Thomas, Meister Michael, Hartmann Arndt, Warth Arne, Agaimy Abbas (2017). SMARCA4 and SMARCA2 deficiency in non-small cell lung cancer: immunohistochemical survey of 316 consecutive specimens. Ann Diagn Pathol.

[ref-14] Agaimy Abbas, Daum Ondrej, Märkl Bruno, Lichtmannegger Ines, Michal Michal, Hartmann Arndt (2016). SWI/SNF Complex-deficient Undifferentiated/Rhabdoid Carcinomas of the Gastrointestinal Tract: A Series of 13 Cases Highlighting Mutually Exclusive Loss of SMARCA4 and SMARCA2 and Frequent Co-inactivation of SMARCB1 and SMARCA2. Am J Surg Pathol.

[ref-15] Ribeiro-Silva Cristina, Vermeulen Wim, Lans Hannes (2019). SWI/SNF: Complex complexes in genome stability and cancer. DNA Repair (Amst).

[ref-16] Schaefer Inga-Marie, Hornick Jason L. (2021). SWI/SNF complex-deficient soft tissue neoplasms: An update. Semin Diagn Pathol.

[ref-17] Schwartz Christopher J., Pareja Fresia, Silva Edaise M., Selenica Pier, Ross Dara S., Weigelt Britta, Brogi Edi, Reis-Filho Jorge S., Wen Hannah Y. (2021). Histologic and genomic features of breast cancers with alterations affecting the SWI/SNF (SMARC) genes. Mod Pathol.

[ref-18] Agaimy Abbas, Bishop Justin A. (2021). SWI/SNF-deficient head and neck neoplasms: An overview. Semin Diagn Pathol.

[ref-19] Ramalingam Preetha, Croce Sabrina, McCluggage W. Glenn (2017). Loss of expression of SMARCA4 (BRG1), SMARCA2 (BRM) and SMARCB1 (INI1) in undifferentiated carcinoma of the endometrium is not uncommon and is not always associated with rhabdoid morphology. Histopathology.

[ref-20] Nambirajan Aruna, Singh Varsha, Bhardwaj Nishu, Mittal Saurabh, Kumar Sunil, Jain Deepali (2021). SMARCA4/BRG1-Deficient Non-Small Cell Lung Carcinomas: A Case Series and Review of the Literature. Arch Pathol Lab Med.

